# Ablation of spinal cord estrogen receptor α‐expressing interneurons reduces chemically induced modalities of pain and itch

**DOI:** 10.1002/cne.24847

**Published:** 2020-01-06

**Authors:** May Tran, Joao Manuel Braz, Katherine Hamel, Julia Kuhn, Andrew J. Todd, Allan I. Basbaum

**Affiliations:** ^1^ Department of Anatomy University of California San Francisco California; ^2^ Spinal Cord Group, Institute of Neuroscience and Psychology, College of Medical, Veterinary and Life Sciences University of Glasgow Glasgow UK

**Keywords:** estrogen receptor, excitatory interneurons, itch, pain, spinal cord

## Abstract

Estrogens are presumed to underlie, at least in part, the greater pain sensitivity and chronic pain prevalence that women experience compared to men. Although previous studies revealed populations of estrogen receptor‐expressing neurons in primary afferents and in superficial dorsal horn neurons, there is little to no information as to the contribution of these neurons to the generation of acute and chronic pain. Here we molecularly characterized neurons in the mouse superficial spinal cord dorsal horn that express estrogen receptor α (ERα) and explored the behavioral consequences of their ablation. We found that spinal ERα‐positive neurons are largely excitatory interneurons and many coexpress substance P, a marker for a discrete subset of nociceptive, excitatory interneurons. After viral, caspase‐mediated ablation of spinal ERα‐expressing cells, we observed a significant decrease in the first phase of the formalin test, but in male mice only. ERα‐expressing neuron‐ablation also reduced pruritogen‐induced scratching in both male and female mice. There were no ablation‐related changes in mechanical or heat withdrawal thresholds or in capsaicin‐induced nocifensive behavior. In chronic pain models, we found no change in Complete Freund's adjuvant‐induced thermal or mechanical hypersensitivity, or in partial sciatic nerve injury‐induced mechanical allodynia. We conclude that ERα labels a subpopulation of excitatory interneurons that are specifically involved in chemically evoked persistent pain and pruritogen‐induced itch.

## Graphical abstract

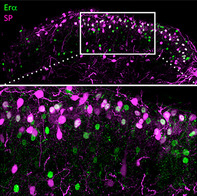

Estrogens underlie, at least in part, the greater pain sensitivity and chronic pain prevalence that women experience compared to men. Previous studies revealed populations of estrogen receptor‐expressing neurons in primary afferents and in superficial dorsal horn neurons, but there is limited information as to the contribution of these neurons to the generation of acute and chronic pain. Here we molecularly characterized neurons in the mouse superficial spinal cord dorsal horn that express estrogen receptor α (ERα) and explored the behavioral consequences of their ablation. The great majority of spinal ERα‐positive neurons are excitatory interneurons and many co‐express the neuropeptide, substance P. After viral, caspase‐mediated ablation of spinal ERα‐expressing cells, we did not observe changes in acute mechanical or heat pain processing, but recorded significant changes in the response to chemically‐evoked persistent pain behavior as well as pruritogen‐evoked itch.

## 1 INTRODUCTION

Many chronic pain conditions, notably migraine, fibromyalgia, and temporomandibular joint disorders are more common in women than in men (Berkley, 1997; Unruh, 1996). This sex difference only becomes apparent at puberty and diminishes after menopause (Brandes, 2006; LeResche, 1997). Women are also more sensitive than men on measures of acute pressure, electrical, heat, and cold pain (Fillingim, King, Ribeiro‐DaSilva, Rahim‐Williams, & Riley III, 2009). It is very likely, therefore, that estrogen, the primary female sex hormone, contributes to pain processing. Estrogens bind to a number of receptors, notably estrogen receptor α and β (ERα and ERβ) and to a G‐protein‐coupled estrogen receptor, GPER (Prossnitz & Barton, 2011; Toran‐Allerand, 2005). Estrogen receptors are expressed throughout the body, including the ovaries, testes, liver, lungs, and brain (Couse, Lindzey, Grandien, Gustafsson, & Korach, 1997). As a result, estrogen can influence functions as diverse as sexual development, immune regulation, and memory (McEwen & Alves, 1999).

With respect to pain circuitry, estrogen receptors are expressed both in sensory neurons and in interneurons of the spinal and medullary dorsal horns (Amandusson, Hermanson, & Blomqvist, 1995; Papka, Srinivasan, Miller, & Hayashi, 1997; Shughrue, Lane, & Merchenthaler, 1997; Vanderhorst Veronique, Gustafsson, & Ulfhake, 2005). Recently, we reported that aromatase, the enzyme that catalyzes the conversion of testosterone to estradiol is expressed in inhibitory neurons of laminae I and V of the spinal and medullary dorsal horns. We suggested that in addition to circulating estrogen, spinal and medullary‐derived estrogen may also engage nociceptive circuits (Tran, Kuhn, Braz, & Basbaum, 2017). Whether an interaction of estrogen with these different populations of estrogen receptor‐expressing neurons exerts comparable or differential effects is unclear. Furthermore, as estrogen appears to be pronociceptive in some reports, for example, in a behavioral model of mechanically‐induced visceral pain (Ji, Tang, & Traub, 2011), but antinociceptive in other pain settings (Craft, 2007), contradictory conclusions have been drawn as to the estrogen contribution to pain processing. In addition, as yet, the contribution of spinal ERα to somatic pain processing in general, or to specific pain modalities, for example, heat versus mechanical pain, has not been reported.

Although studies using knockout mice, pharmacology, gonadectomy, and other hormonal manipulations have made valuable contributions to our understanding of estrogenic function in both health and disease (Couse & Korach, 1999; Paterni, Granchi, Katzenellenbogen, & Minutolo, 2014), these approaches affect estrogen activity on a global level, making it difficult to establish regional functional specificity in the contribution of estrogens (Amandusson & Blomqvist, 2013; Greenspan et al., 2007). Here, we specifically addressed the contribution of ERα‐expressing dorsal horn neurons to different modalities of pain and itch. We first determined the extent to which these neurons express markers of excitatory or inhibitory interneurons. Next, we used a Cre‐dependent viral strategy to ablate ERα^+^‐expressing cells in the spinal cord dorsal horn of adult mice and evaluated responses to a variety of mechanical, thermal, chemical, and pruritic stimuli. We show that the ERα‐expressing neurons in the spinal cord dorsal horn are predominantly excitatory interneurons. Their ablation led to a selective reduction in formalin‐ and histamine‐induced behaviors, suggesting that the ERα‐expressing neurons comprise a functionally distinct subset of excitatory interneurons that mediate chemical pain and pruritogen‐induced itch.

## 2 MATERIALS AND METHODS

### 2.1 Mouse lines

All experiments were approved by and performed according to the guidelines of the University of California, San Francisco's Institutional Animal Care and Use Committee. For ERα cell ablation experiments, we used ERα‐Cre mice, which are mice heterozygous for Cre recombinase that was knocked into the locus of the *Esr1* gene in a manner that preserves expression of ERα (Lee et al., 2014). We used their wildtype littermates (ERα‐WT) as controls. TR4 mutant mice were generated as previously described (Wang et al., 2013).

### 2.2 Viral injections for ablation and knockout

Spinal injection of virus was performed as previously described (Bráz et al., 2012). In brief, we anesthetized mice with ketamine/xylazine (60 and 8.0 mg/kg) and then made a dorsal laminectomy to expose the left side of the lumbar enlargement. Using a micropipette attached to a stereotaxic instrument‐mounted microinjector, we made multiple injections of virus, rostrocaudally along two segments of the lumbar enlargement. Each mouse received a total of 2.0 μl of viral stock solution; each injection contained up to 200 nl. For the ERα‐Cre cell ablation experiments, we injected AAV1‐flex‐taCasp3‐TEVp (caspase virus, titer: 1.5–2.8 × 10^12^ viral particles/ml; Gene Therapy Vector Core at the University of North Carolina at Chapel Hill and Dr. R. Jude Samulski; Yang et al., 2013) into ERα‐Cre mice and wildtype littermate controls.

### 2.3 Viral injections for neuroanatomical characterization

In a previous study, we injected a Cre‐dependent EGFP reporter virus (AAV1‐FLEX‐eGFP) into the spinal cord of Tac1‐Cre mice (Gutierrez‐Mecinas et al., 2017) to characterize the distribution of Substance P‐expressing interneurons in the dorsal horn. Here we immunostained spinal cord tissue from these animals for expression of ERα and evaluated overlap with GFP.

### 2.4 Behavioral tests

For all behavioral testing and scoring, the experimenter was blind to mouse genotype. Mice were tested in a first session prior to caspase virus injection to measure baseline thresholds and again 3 weeks after virus injection to measure post‐virus thresholds. For studies examining chronic pain models (see below), mice were also tested post‐tissue or nerve injury.

### 2.5 Mechanical threshold

Mice were placed into individual acrylic cylinders on a wire mesh and allowed to acclimate for 1–2 hr. Withdrawal responses to von Frey filaments (North Coast Medical, Gilroy, CA) applied to the plantar surface of the left hindpaw were recorded and mechanical thresholds were calculated using the up‐down method (Chaplan, Bach, Pogrel, Chung, & Yaksh, 1994).

### 2.6 Thermal threshold

Mice were placed into individual chambers inside acrylic boxes on a 25.0°C heated glass surface of a thermal nociception test device (Dirig, Salami, Rathbun, Ozaki, & Yaksh, 1997; Hargreaves, Dubner, Brown, Flores, & Joris, 1988) and allowed to acclimate for 1–2 hr. Radiant heat intensity was set to 65 units (current output: 4.2–4.5 A) and then the light source was positioned to stimulate the plantar surface of the left hindpaw. Withdrawal latencies to the infrared light were recorded up to a cutoff of 20 s.

### 2.7 Capsaicin and formalin test

For capsaicin and formalin tests, the mice were placed into individual acrylic cylinders on a glass surface on top of an angled mirror and allowed to acclimate for 30 min. Mice were then lightly restrained with a towel and then capsaicin (Sigma‐Aldrich, St. Louis, Missouri; 3 μg in 10 μl of 10% ethanol, 10% Tween‐80, 80% saline) or formalin (10 μl of 2% solution made by diluting 37% formaldehyde 1/50 in saline; ACROS Organics, Morris Plains, NJ) was injected into the plantar surface of the left hindpaw with a 100 μl‐capacity Hamilton syringe (Hamilton Company, Reno, NV) fitted with a 30‐gauge needle. Mice were immediately returned to the cylinders and video recorded for 5 min (capsaicin) or 1 hr (formalin). Behavior was scored as time spent licking and/or biting the left hindpaw. Formalin behavior was separated into three distinct phases: phase I was defined as the first 0–5 min following the injection, interphase as the period 5–10 min after the injection, and phase II lasted from 10 to 60 min postinjection.

### 2.8 Tests of pruritoception

To distinguish itch from pain after an injection into the hindlimb, we followed the protocol of LaMotte, Shimada, and Sikand (2011), in which an algogen and pruritogen, respectively, provoke licking and biting of the injected region. Using the same cylinders, we made a subcutaneous injection of 100 μl of either chloroquine (200 μg diluted in saline; Sigma‐Aldrich) or histamine (500 μg diluted in saline; Sigma‐Aldrich) into the left calf (Akiyama, Nagamine, Carstens, & Carstens, 2014; LaMotte et al., 2011). Mice were immediately returned to the cylinders and video recorded for 30 min. Behavior was scored as time spent licking and/or biting the injection area.

### 2.9 Tissue injury‐induced chronic pain: Complete Freund's adjuvant

To induce prolonged inflammation, we injected complete Freund's adjuvant (CFA; Sigma‐Aldrich; 20 μl of 1:1 emulsion in saline) into the plantar surface of the left hindpaw of mice lightly restrained with a towel. Three to 4 days later, when animals display significant paw edema and hypersensitivity (Malmberg, Gilbert, McCabe, & Basbaum, 2003), we used the von Frey and Hargreaves tests to measure mechanical and thermal (heat) thresholds.

### 2.10 Neuropathic pain: Sciatic nerve injury

To model neuropathic pain, we performed sciatic nerve injury (SNI) as described previously (Shields, Eckert III, & Basbaum, 2003). Briefly, under 2% isoflurane anesthesia, we exposed the sciatic nerve, and then ligated and excised 2.0 mm of the peroneal and sural branches, sparing the tibial branch. The incision was then sutured closed and the mice were allowed to recover and returned to their home cages. One and 7 days later, when animals display significant hypersensitivity, mechanical thresholds were measured.

### 2.11 Retrograde tracing

Under ketamine/xylazine anesthesia, mice were placed in a stereotaxic apparatus (Kopf Instruments, Tujunga, CA) and 0.5–1.0 μl of Fluorogold (Fluorochrome, Denver, CO) was injected into the left lateral parabrachial nucleus, according to coordinates from Paxinos and Franklin's *The Mouse Brain in Stereotaxic Coordinates*. Animals were perfused 7 days later and tissue was processed for immunohistochemistry.

### 2.12 Immunohistochemistry

To determine marker overlap and for viral tracing experiments, we performed fluorescent immunohistochemistry. Mice received an intraperitoneal injection of 2.5% Avertin (2,2,2‐Tribromoethanol, Sigma‐Aldrich) and were transcardially perfused with 10 ml of phosphate buffered saline (PBS) followed by 30 ml of 10% formalin in PBS. Spinal cord and dorsal root ganglia (DRGs) were dissected out and postfixed in 10% formalin in PBS overnight. Tissue was then cryoprotected in 30% sucrose overnight. For immunostaining, spinal cord (25 μm) and DRG (14 μm) sections were cut on a cryostat, mounted on slides and blocked for 1 hr in 10% normal goat serum in PBS containing 0.3% Triton X‐100. Primary antibody incubation was done overnight at room temperature (RT). Table 1 provides details of the primary antibodies used. The following day, after three PBS washes, the sections were incubated in secondary antibodies for a minimum of 2 hr at RT. Secondary antibodies were Alexa Fluor 488, 594, or 647 raised in goat (Thermo Fisher Scientific, Waltham, MA) and used at 1:1000 in PBS. Following three final washes with PBS, the slides were allowed to dry and then coverslipped using Fluoromount‐G aqueous mounting medium (SouthernBiotech, Birmingham, Alabama). Tissue from the AAV1‐FLEX‐eGFP‐injected Tac1‐Cre mice was postfixed for 2 hr and cut at 60 μm on a vibrating blade microtome. Primary antibody incubations were performed at 4°C. After completion of behavioral testing, the animals were perfused for immunohistochemistry to quantify numbers of ERα^+^ cells remaining in the lumbar spinal cord dorsal horn. We did not perform fluorescent immunohistochemistry because debris from the injection was highly autofluorescent, making debris difficult to distinguish from ERα + immunoreactivity. The DAB immunohistochemistry was performed following the protocol of Llewellyn‐Smith, DiCarlo, Collins, and Keast (2005). First, to remove endogenous peroxidase activity, we incubated slides at RT in methanol peroxide (1% hydrogen peroxide, 30% methanol, diluted in water) for 30 min. The slides were then washed three times for 10 min each in 10 mM TRIS base (Trizma, Sigma‐Aldrich) and 0.05% merthiolate (Thimerosal, Sigma‐Aldrich) in 10 mM phosphate buffer, pH 7.4 (TPBS) that also contained 0.3% Triton X‐100 (TPBS + Triton = immunobuffer, IB). Sections were then blocked for a minimum of 30 min in 10% normal horse serum (NHS) in IB followed by overnight RT incubation in ERα primary antibody (1:20,000in 10% NHS in IB; rabbit, Millipore, 06‐935). The next day, the slides were washed three times for 10 min each time in TPBS and then incubated overnight at RT in biotin‐SP‐conjugated donkey anti‐rabbit secondary antibody (Jackson ImmunoResearch, West Grove, Pennsylvania, United State; diluted 1:500 in 1% NHS in IB). The following day, the slides were washed three times for 10 min each in TPBS and then incubated for a minimum of 4 hr in ExtrAvidin‐Peroxidase (Sigma‐Aldrich; diluted 1:1500 in IB). The sections were subsequently washed three times for 10 min each time in TPBS and then incubated for 10 min in a solution of 0.004% ammonium chloride, 0.2% D‐glucose, 0.04% nickel ammonium sulfate, and 0.5 mg/mL 3,3′diaminobenzidine tetrahydrochloride in 10 mM PB, pH 7.4. An equal volume of the same buffer, but containing 2.0 μl/ml of glucose oxidase was then added to the slides to yield a final concentration of 1.0 μl/ml, glucose oxidase. After 8 min, we stopped the reaction by rapidly rinsing the slides six to seven times in TPBS, followed by three to four rinses with distilled water. After drying at RT for several hours, the sections were cleared by washing twice in xylene and coverslipped using Permaslip mounting medium (Alban Scientific, St. Louis, MO).

**Table 1:** Primary antibodies used for immunohistochemistry

Antibody	Manufacturer	Cat #	Species	Concentration	RRID
ERα	Millipore	06‐935	Rabbit	1:10,000‐20,000	AB_310305
NeuN	Millipore	MAB377	Mouse	1:5000	AB_2298772
PKCγ	Strategic BioSolutions	Gift	Guinea‐pig	1:5000	
Calretinin	Swant	6B3	Mouse	1:5000	AB_10000320
GFP	Abcam	ab13970	Chicken	1:2000	AB_30798
Pax2	Abnova	H00005076‐M01	Mouse	1:2000	

### 2.13 Antibody characterization

Table 1 lists all antibodies used in this study. Rabbit anti ERα detects in Western blots a roughly 58 kDa band from MCF7 cell lysate (manufacturer's information) and a 55 kDa band from cichlid whole brain extract (Munchrath & Hofmann, 2010). Preincubation with the antigen eliminates all bands (Friend, Resnick, Ang, & Shupnik, 1997). In addition, there was no detectable signal in spinal cord tissue immunostained from ERα conditional knockout mice (unpublished observation). Mouse anti NeuN recognizes neuronal nuclei and cytoplasm. Antibody specificity has been evaluated with immunohistochemistry and immunoblot analysis, showing that immunoreactivity is present only in neurons (Mullen, Buck, & Smith, 1992). Anti‐PKCγ antibodies were raised in guinea pigs and when used in formaldehyde‐fixed animals generated the following pattern of spinal cord immunostaining: dense immunoreactivity in lamina IIi and the corticospinal tract of wildtype mice. This pattern is in complete agreement with our previous studies using rabbit‐generated PKCγ antibodies that did not immunostain the spinal cord or brain of PKCγ mutant mice (Malmberg, Chen, Tonegawa, & Basbaum, 1997). Anti‐calretinin antibodies were produced in mice by immunization with recombinant human calretinin‐22 k, an alternative splice product of the calretinin gene and identical with calretinin up to Arg178. The antibody 6B3 recognizes an epitope within the first four EF‐hands domains common to both calretinin and calretinin‐22 k. This antibody does not cross‐react with calbindin D‐28 k or other known calcium binding‐proteins and does not immunostain the brain of calretinin mutant mice (manufacturer's specifications). The pattern of Rabbit anti‐Pax2 immunostaining that we observe completely agrees with previous reports that characterized spinal cord dorsal horn Pax2‐expressing cells as inhibitory interneurons (Kardon et al., 2014; Punnakkal, von Schoultz, Haenraets, Wildner, & Zeilhofer, 2014). In addition, for this particular antibody, Western blot from human fetal kidney tissue recognizes a band at the proper expected size of 45 kDa (manufacturer's information). Anti‐GFP antibodies were raised in chicken against the recombinant full‐length protein corresponding to GFP. Our own studies have established that there is no GFP immunoreactivity in wildtype mice (Braz, Enquist, & Basbaum, 2009).

### 2.14 Imaging and quantification

Immunofluorescent tissue samples were imaged with ZEN 2010 software (Zeiss) in a LSM 700 confocal microscope (Zeiss, Oberkochen, Germany) using a ×20 objective. Images of 3–6 randomly selected spinal cord sections from each mouse were acquired and processed in Fiji/ImageJ (NIH), which involved cropping, assigning colors to individual channels, brightness and contrast adjustment, maximum intensity projections of Z‐stacks, and quantification. For quantification, the Isodata Threshold algorithm was used to define labeled cells in each channel and the Particle Analyzer tool (size range: 15–150 μm, circularity: 0.5–1) was used to count cells. An overlay of the channels was then used to distinguish double‐labeled cells. Any changes to brightness and contrast were applied uniformly within a single image and across images in the same experiment.

To count cells in the ablation experiments, the slides were automatically scanned with a ×20 objective under brightfield conditions using a Zeiss Axio Scan.Z1 slide scanner. Images were stitched with Zeiss ZEN2 software and then exported to FIJI/ImageJ. The images were converted to 8‐bit grayscale and then Brightness/Contrast was modified using the “Auto” feature. Images were then cropped to display only the area from the central canal to the dorsal border of the tissue. To distinguish one side of the spinal cord from the other, we drew a perpendicular line from the central canal to the dorsal border of the cord. Using the Cell Counter tool, an observer blinded to mouse genotype manually counted ERα^+^ cells on each side of the cord. To calculate the percentage of ERα^+^ cells remaining after virus injection, we divided the number of ERα^+^ cells on the ipsilateral side by the number of cells on the contralateral side. For our ERα cell ablation experiments, we set a threshold of 25% for ablation; that is, if an ERα‐Cre mouse had less than 25% of ERα cells remaining, we considered it to be a successful ablation and included data generated from this mouse in our analysis, but if more than 25% of cells remained, data from this mouse were excluded. Conversely, for ERα‐WT control mice, if fewer than 50% of cells remained, the data were excluded.

### 2.15 Experimental design and statistical analysis

Statistical analyses were chosen in consultation with the University of California, San Francisco's Clinical and Translational Science Institute. To compare anatomical results between male and female mice, we used Unpaired (Student's) *t* tests, provided that the data were normally distributed (Shapiro–Wilk test) and demonstrated homogeneity of variances (*F* test). If groups had unequal variances, we used unpaired *t* tests with Welch's correction. If the groups were not normally distributed, we used the Mann–Whitney *U* test.

For each behavioral test, the results were unblinded and grouped by two factors: sex (male, female) and genotype (ERα‐Cre or ERα‐WT). Each of the four resulting groups was then tested for normality using the Shapiro–Wilk test. If data were not normally distributed, all four groups were log transformed to normalize the data so that data would fulfill the requirements for analysis with two‐way analysis of variance (ANOVA). The following data sets underwent log transformation: ERα cell ablation mechanical threshold, ERα cell ablation capsaicin, ERα cell ablation formalin interphase, ERα cell ablation formalin phase II, and ERα cell ablation chloroquine. In the case of ERα cell ablation formalin interphase, prior to log transformation, data were translated by adding one to all data points because certain scores had a value of 0. All data sets demonstrated homogeneity of group variances as assessed by Levene's test. The Shapiro–Wilk test and Levene's test were performed in Microsoft Excel 2011 using the Real Statistics Resource Pack for Mac (Release 3.5.3), copyright 2013–2017 by Charles Zaiontz, www.real-statistics.com. Data were next transferred to GraphPad Prism (version 6.0h for Mac) for two‐way ANOVA with Sidak's multiple comparisons test. We set up two comparisons: ERα‐WT males versus ERα‐Cre males and ERα‐WT females versus ERα‐Cre females. Statistical significance is indicated in the figure legends. For nonnormal data sets, transformed data were used for statistical analysis, but raw data were used in graphs for ease of comprehension. In the experiments where data from male and female were pooled due to low numbers of subjects with successful ablation, we applied *t* tests or Mann–Whitney *U* tests under the same guidelines described for the anatomical studies.

## 3 RESULTS

*Neurochemical characterization of ERα‐expressing cells in the spinal cord dorsal horn*.

**Figure 1:**
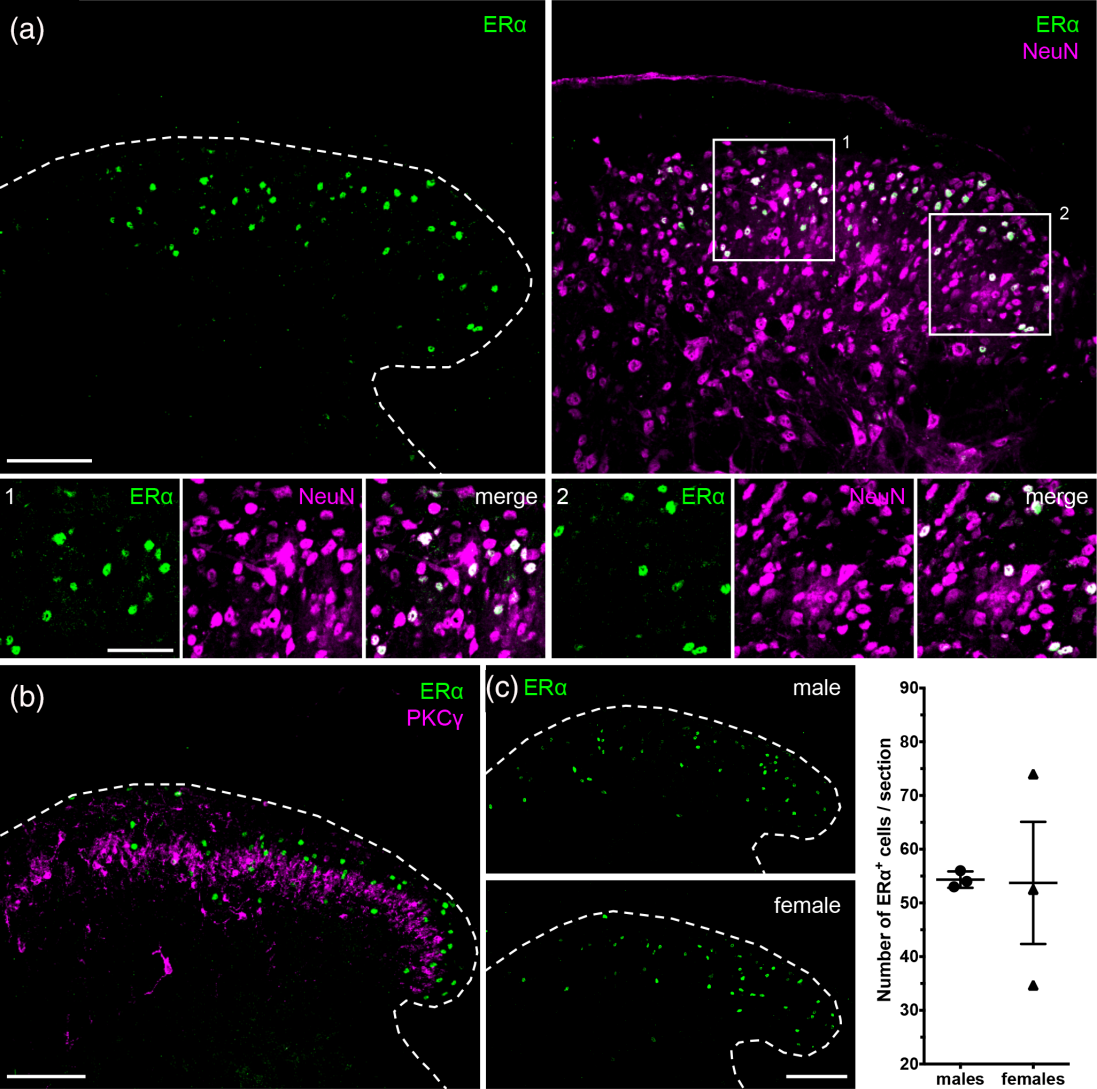
Estrogen receptor α (ERα) expression in the spinal cord. (a) ERα (green) is expressed by NeuN (magenta) positive neurons in the spinal cord dorsal horn. Insets 1 and 2 depict examples of overlap. (b) ERα is mainly expressed by cells of lamina II of the dorsal horn, with scattered cells both superficially and in deeper laminae (III–V). A subset of PKCγ excitatory interneurons serves as a landmark for inner lamina II. ERα and PKCγ do not overlap. (c) Males and females have comparable numbers of dorsal horn ERα^+^ cells. Right panel displays quantification from three male and three female mice. Data are presented as mean ± *SEM* (males: 54 ± 0.88 and females: 54 ± 11). Two‐tailed unpaired *t* test with Welch's correction for unequal variances: *t* = 0.05349, df = 2, *p* = .9622. Dashed lines outline the border of the spinal cord dorsal horn. Scale bar: 100 μm; inset: 50 μm [Color figure can be viewed at wileyonlinelibrary.com]

ERα‐immunoreactive cells are concentrated in superficial laminae (Figure 1a, left panel) of the spinal cord dorsal horn and express the neuronal marker NeuN (Figure 1a, right panel). Based on the distribution of PKCγ‐expressing excitatory interneurons, which mark inner lamina II (lamina II_i_; Figure 1b), it is apparent that the majority of ERα‐expressing cells are in outer lamina II (lamina II_o_), with some cells in lamina II_i_ and a few in lamina I and in deeper laminae. Furthermore, we found no sex differences in either the number or the distribution pattern of spinal ERα^+^ cells (Figure 1c).

**Figure 2:**
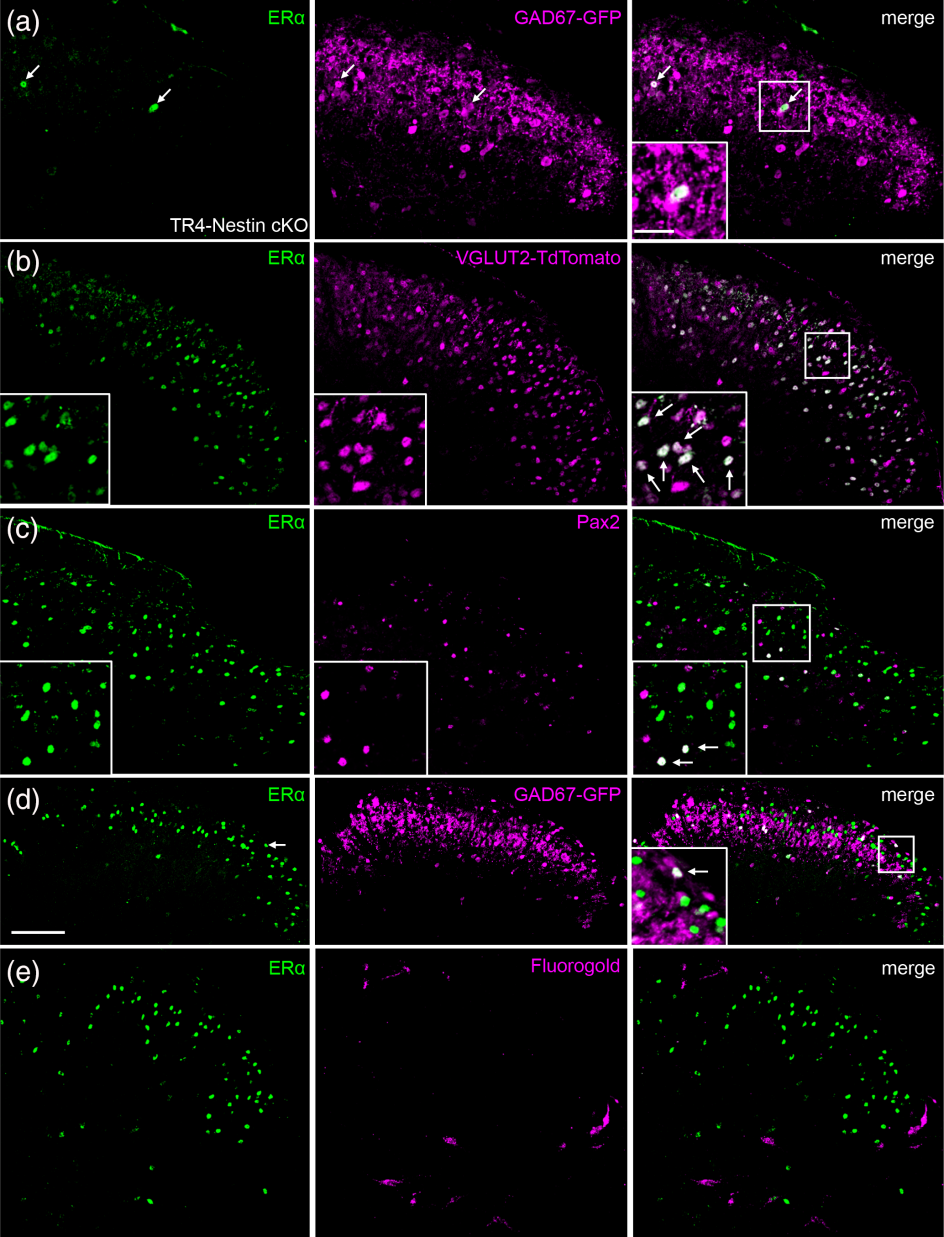
ERα predominates in excitatory interneurons. (a) Very few neurons express ERα in the spinal cord of TR4 knockout mice, which lack large numbers of excitatory interneurons. The remaining few ERα^+^ cells are likely inhibitory, as determined by colocalization with GAD67‐GFP. (b,c) The great majority of ERα‐expressing cells overlap with TdT‐Tomato in a VGLUT2‐Td‐Tomato reporter mouse (b), but not with Pax2 (c), a marker of spinal cord inhibitory interneurons. (d) Consistent with this conclusion, only a small number of ERα‐expressing cells coexpress GFP in the spinal cord of the GAD67‐GFP reporter mice. (e) ERα‐expressing cells were never retrogradely labeled after injection of the Fluorogold retrograde tracer into the lateral parabrachial nucleus. Arrows in all panels, including insets, point to examples of overlap between ERα and a second marker. Scale bar: 100 μm [Color figure can be viewed at wileyonlinelibrary.com]

We previously reported that TR4‐Nestin knockout mice exhibit an extensive loss of excitatory interneurons in laminae I and II_o_, and that this results in insensitivity to mechanical stimuli as well as to capsaicin and several pruritogens (Wang et al., 2013). A reevaluation of the TR4‐Nestin mice (Figure 2a; left panel) shows that there is also a significant loss of ERα^+^ neurons in the dorsal horn, which suggests that the majority of ERα^+^ neurons are excitatory interneurons. Consistent with this conclusion we found that the majority of ERα^+^ neurons double label with Td‐Tomato in a VGLUT2‐Td‐Tomato reporter mouse (Figure 2b), but not with Pax2, a marker of spinal cord inhibitory interneurons (Cheng et al., 2004; Figure 2c).

Interestingly, the few remaining ERα + cells in the TR4‐Nestin mice colocalize with GAD67‐GFP, a marker of inhibitory cells (Figure 2a). In addition, in spinal cord tissue from GAD67‐GFP reporter mice (Tamamaki et al., 2003), we observed that only 16 ± 5.1% of ERα‐expressing cells are also GAD67‐GFP^+^ (*n* = 1 male, 2 females; Figure 2d), supporting our conclusion that ERα predominates in excitatory interneurons. Finally, when we injected the Fluorogold retrograde tracer into the lateral parabrachial nucleus, a region that receives the overwhelming majority of projection neurons from laminae I and V in the mouse (Cameron et al., 2015), we never observed Fluorogold labeling in ERα^+^ cells (Figure 2e), confirming that ERα^+^ cells are interneurons.

**Figure 3:**
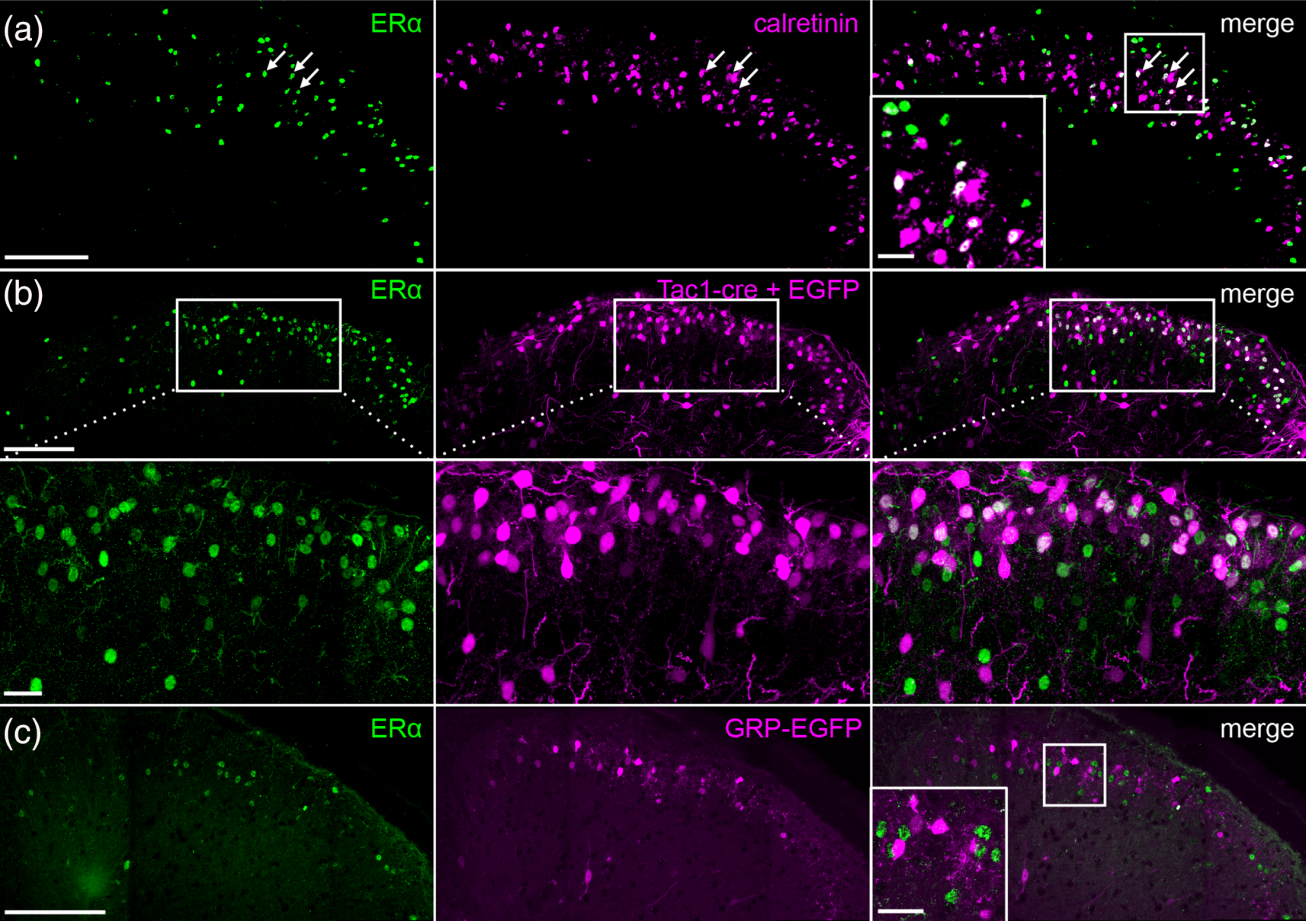
ERα^+^ interneurons coexpress calretinin and substance P, but not GRP. (a) Approximately 40% of ERα‐expressing neurons immunostain for calretinin, a marker of a subset of excitatory interneurons in lamina II. (b) This figure illustrates that injection of Cre‐dependent EGFP reporter virus into the dorsal horn of Tac1‐Cre mice results in considerable coexpression of ERα and GFP, indicating coexpression of ERα and substance P. (c) In contrast, ERα is not present in interneurons marked in a GRP‐EGFP transgenic mouse. Arrows depict examples of overlap between ERα and a second marker. Scale bar: 100 μm; inset: 20 μm [Color figure can be viewed at wileyonlinelibrary.com]

To characterize the subpopulation of ERα + spinal interneurons, we performed a series of double‐immunostaining experiments with known markers of subpopulations of excitatory interneurons. Calretinin marks a large excitatory subpopulation in lamina II, although it also labels a few cells in lamina I and a few inhibitory interneurons (Gutierrez‐Mecinas et al., 2019; Smith et al., 2015). Double immunostaining with ERα revealed that 39 ± 2.2% of ERα‐expressing cells express calretinin (*n* = 2 males, 1 female; Figure 3a). We also examined colabeling for substance P, another marker of dorsal horn excitatory interneurons (Dickie et al., 2019; Gutierrez‐Mecinas et al., 2017; Huang et al., 2019). Here we colabeled for ERα and GFP in a substance P reporter mouse (Tac1‐Cre injected with a Cre‐dependent EGFP reporter virus; Dickie et al., 2019) and found extensive colocalization of EGFP and ERα (Figure 3b). On the other hand, although gastrin‐releasing peptide (GRP) also marks a subset of lamina I‐II excitatory interneurons (Solorzano et al., 2015), we found no overlap with ERα in a GRP‐EGFP reporter line (Figure 3c). Taken together, these data indicate that ERα is primarily associated with distinct subsets of excitatory interneurons.

### 3.1 Nociceptive and pruriceptive behavioral phenotypes after ERα^+^ interneuron ablation

**Figure 4:**
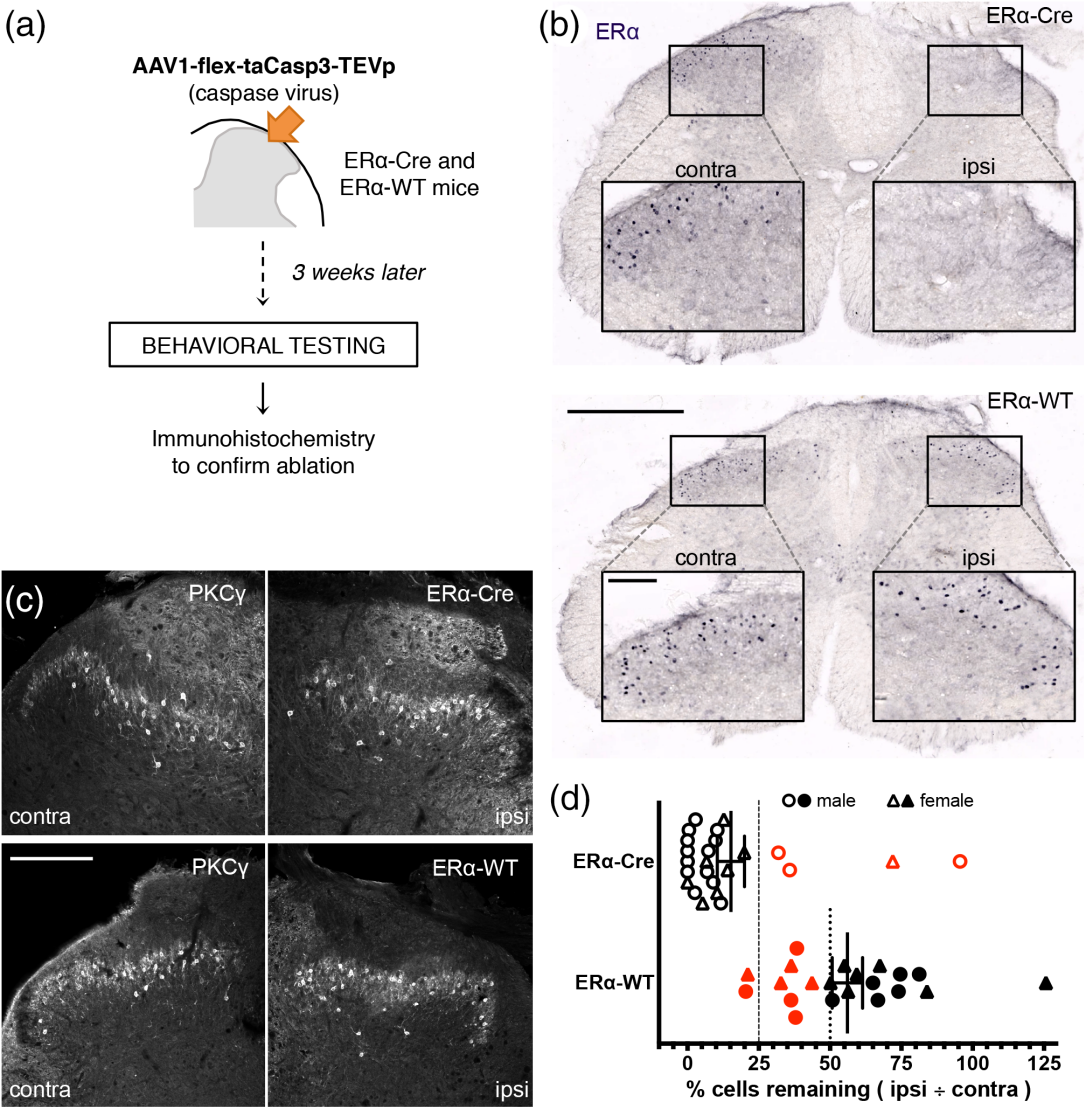
Ablation of ERα‐expressing cells in the spinal dorsal horn. (a) To ablate ERα‐expressing interneurons ERα‐Cre mice received unilateral injections of a Cre‐dependent caspase virus (AAV1‐flex‐taCasp3‐TEVp) into the lumbar dorsal horn. Similarly injected wildtype littermates (ERα‐WT) served as controls. (b) Immunostaining for ERα confirms the ipsilateral ablation of ERα‐expressing neurons (top) and preservation of the cells in the ERα‐WT (bottom) mouse. Scale bar: 500 μm; Insets: 100 μm. (c) Preservation of the PKCγ staining pattern in the ipsilateral dorsal horn confirms that ERα^+^ cell ablation did not induce nonspecific cell death (top right vs. top left panel). Scale bar: 200 μm. (d) Quantification of ERα^+^ cell ablation illustrates the thresholds that were set a priori to determine which behavioral results would be included in subsequent analyses. For ERα‐Cre mice, ablation was considered successful if fewer than 25% ERα^+^ neurons remained (dashed line). For ERα‐WT mice, we excluded any animal that had fewer than 50% of ERα^+^ cells remaining (dotted line). Excluded mice are indicated in red [Color figure can be viewed at wileyonlinelibrary.com]

To assess whether ERα^+^ interneurons contribute to pain and/or itch and to what extent the ERα^+^ interneurons account for the TR4‐Nestin insensitivity phenotype, we ablated the ERα‐expressing neurons in the dorsal horn by local injection of a Cre‐dependent caspase virus (AAV1‐flex‐taCasp3‐TEVp; Yang et al., 2013). Their wildtype littermates served as controls (ERα‐WT; Figure 4a). As expected, we observed clear loss of ERα immunostaining in the dorsal horn ipsilateral to the virus injection in the ERα‐Cre mice, but not in the wild type mice (Figure 4b). Importantly, PKCγ immunostaining was preserved in both the ERα‐Cre and ERα‐WT mice (Figure 4c). We cannot conclude that there was absolutely no “off target” ablation of neurons. However, given the incredible diversity of neurochemical subtypes of spinal cord interneurons, the fact that the PKCγ immunoreactivity persisted after injection of the caspase virus, we are confident that the injection did not indiscriminately result in dorsal horn cell death. Although all mice were examined in the behavioral studies, to include a mouse in the behavioral analysis, we a priori established a minimum requirement of cell ablation. Only mice with < 25% of ERα^+^ cells remaining in the ipsilateral spinal dorsal horn, compared to the contralateral, uninjected side in an ERα‐Cre mouse, were included. We also considered an ERα‐WT mouse with less than 50% of ERα^+^ cells remaining in the ipsilateral dorsal horn, compared to the contralateral, uninjected side, to have received nonspecific damage from the injection and excluded that mouse (Figure 4d). Taken these thresholds into consideration, only 4 of 20 virus‐injected mice included in the less than 90% ablation of ERα‐expressing dorsal horn neurons (Figure 4d).

**Figure 5:**
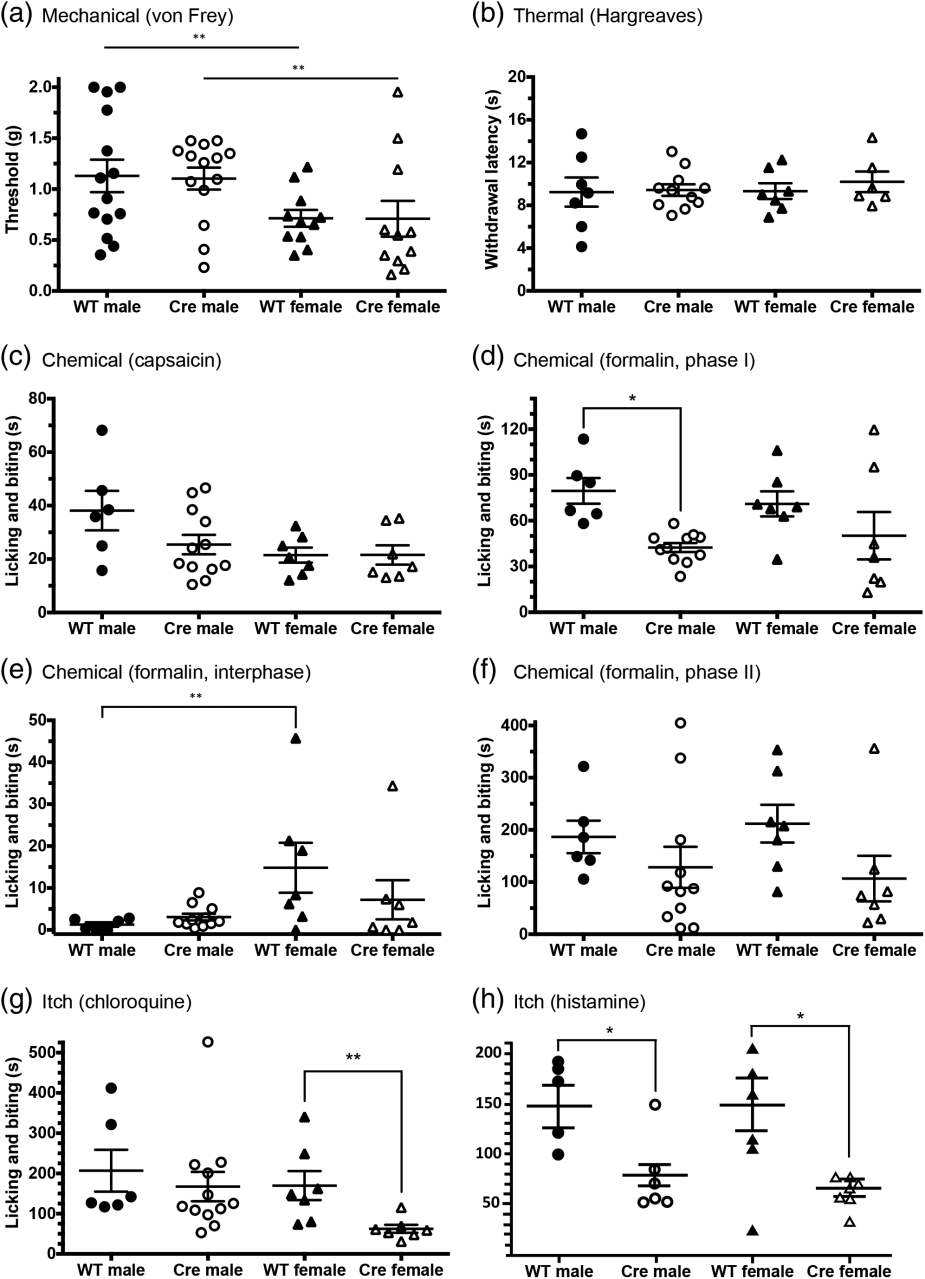
Acute pain and itch responsiveness after ablation of spinal ERα^+^ neurons. (a) Mechanical thresholds (von Frey test) in the ERα‐WT and ERα‐Cre ablated mice did not differ. However, sex accounted for a significant source of variation, with female thresholds lower than male thresholds in both Cre and WT groups (see also Table 2). (b) There is no effect of spinal ERα ablation in the Hargreaves test. (c) Spinal cord ERα ablation did not alter intraplantar capsaicin‐induced licking and biting of the hindpaw. (d) Nocifensive behavior (licking and biting of the hindpaw) in the first phase of the formalin test was significantly decreased in male mice after ablation of ERα^+^ cells (ERα‐Cre male) compared to control males (ERα‐WT male). Females do not display a significant difference after ablation. (e) In the formalin test, we did not record significant differences following ERα cell ablation between the ablated and control groups, males exhibited reduced licking and biting during the interphase compared to females (see also Table 2). (f) Similarly, although there was no statistical significant differences between the ablated and control groups during the second phase of the formalin test, genotype (Cre vs. WT) was a significant source of variation, with ablated mice having lower licking and biting times compared to WT mice (see Table 2). (g) Licking and biting in response to chloroquine injected into the thigh/calf are significantly decreased in ERα‐Cre ablated females, compared to ERα‐WT females. ERα ablation did not have a significant effect in males. (h) In contrast, licking and biting in response to histamine injected into the thigh/calf was reduced by spinal ERα ablation in both male and female mice. Data are presented as mean ± *SEM*, with *p < .05 and **p < .01

Three weeks after virus injection, the mice underwent a battery of nociceptive and pruriceptive behavioral tests (Figure 5 and Table 2). Surprisingly, we found few statistically significant differences between ablated (ERα‐Cre) and control (ERα‐WT) mice. Although we confirmed the presence of baseline differences in mechanical (von Frey) thresholds of male and female mice (Mogil et al., 2006), after ERα neuron ablation we found no differences in mechanical thresholds (Figure 5a), in the Hargreaves test of thermal (heat) threshold (Figure 5b) or in capsaicin‐induced nocifensive behaviors (Figure 5c). ERα‐ablated males, but not females, did show significantly reduced nocifensive behaviors in the first phase of the formalin test (Figure 5d), which is a test of acute chemical pain. There was no difference in the interphase, a period of reduced behavior that precedes the second phase (Figure 5e). On the other hand, neither male nor female ERα‐ablated mice differed from wild type controls in nocifensive behavior during the second phase, which in some respects is a model of postoperative pain (Figure 5f). With respect to responsiveness to pruritogens, we found that female, but not male ERα‐ablated mice were less responsive to chloroquine (Figure 5g). Surprisingly, however, both male and female ERα‐ablated mice were less responsive to histamine (Figures 5h).

**Table 2:** Nociceptive behavior in mice after ablation of spinal ERα + neurons

Behavioral test	Erα‐WT male	Erα‐Cre male	Erα‐WT female	Erα‐Cre female	Sex male vs. female	Genotype Erα‐WT vs. Erα‐Cre	Interaction	
**Mechanical (g) (von Frey)**	1.13 ± 0.16 (*N* = 14)	1.10 ± 0.11 (*N* = 14)	0.71 ± 0.08 (*N* = 11)	0.71 ± 0.18 (*N* = 11)	*F*_(1,46)_ = 8.921 *p* = .004	*F*_(1,46)_ = 0.397 *p* = .532	*F*_(1,46)_ = 0.513 *p* = 0.478	
Sidak's multiple comparisons test	M: WT vs. Cre *p* = .998 F: WT vs. Cre *p* = .607							
**Thermal (s) (Hargreaves)**	9.24 ± 1.37 (*N* = 7)	9.43 ± 0.54 (*N* = 11)	9.33 ± 0.74 (*N* = 7)	10.2 ± 0.96 (*N* = 6)	*F*_(1,27)_ = 0.228 *p* = .637	*F*_(1,27)_ = 0.348 *p* = .560	*F*_(1,27)_ = 0.145 *p* = 0.706	
Sidak's multiple comparisons test	M: WT vs. Cre *p* = .984 F: WT vs. Cre *p* = .775							
**Capsaicin (s)**	38.12 ± 7.39 (*N* = 6)	25.43 ± 3.63 (*N* = 12)	21.48 ± 2.81 (*N* = 7)	21.54 ± 3.63 (*N* = 7)	*F*_(1,28)_ = 3.865 *p* = .059	*F*_(1,28)_ = 1.723 *p* = .200	*F*_(1,28)_ = 1.396 *p* = .247	
Sidak's multiple comparisons test	M: WT vs. Cre *p* = .151 F: WT vs. Cre *p* = .995							
**Formalin phase I (s)**	79.55 ± 8.43 (*N* = 6)	42.40 ± 2.98 (*N* = 11)	71.03 ± 8.22 (*N* = 7)	50.17 ± 15.6 (*N* = 7)	*F*_(1,27)_ = 0.0017 *p* = .967	*F*_(1,27)_ = 10.38 *p* = .003	*F*_(1,27)_ = 0.818 *p* = .374	
Sidak's multiple comparisons test	M: WT vs. Cre *p* = .012 F: WT vs. Cre *p* = .229							
**Formalin interphase (s)**	1.29 ± 0.54 (*N* = 6)	3.08 ± 0.79 (*N* = 11)	14.83 ± 5.95 (*N* = 7)	7.19 ± 4.67 (*N* = 7)	*F*_(1,27)_ = 5.226 *p* = .030	*F*_(1,27)_ = 0.229 *p* = .636	*F*_(1,27)_ = 4.310 *p* = .047	
Sidak's multiple comparisons test	M: WT vs. Cre *p* = .446 F: WT vs. Cre *p* = .171							
**Formalin phase II (s)**	186.5 ± 31.13 (*N* = 6)	128.2 ± 39.37 (*N* = 11)	211.9 ± 36.15 (*N* = 7)	106.7 ± 43.6 (*N* = 7)	*F*_(1,27)_ = 0.0055 *p* = .941	*F*_(1,27)_ = 7.772 *p* = .0096	*F*_(1,27)_ = 0.043 *p* = .837	
Sidak's multiple comparisons test	M: WT vs. Cre *p* = .139 F: WT vs. Cre *p* = .095							
	**Erα‐WT**		**Erα‐Cre**		**Two‐tailed unpaired *t* test** **or Mann–Whitney *U* test**				
**CFA mechanical (g)**	0.024 ± 0.01 *N* = 3 males and 3 females		0.055 ± 0.04 *N* = 1 male and 3 females		*t*_(8)_ = 0.9811 *p* = .3553				
**CFA thermal (s)**	6.38 ± 1.52 *N* = 3 males and 4 females		4.59 ± 0.53 *N* = 1 male and 3 females		*U* = 12 *p* = .7879				

*Note*: Data are presented as mean ± *SEM*; number of animals in each group (and sex, if applicable) is reported for each behavioral test. Units of measure for the von Frey data are threshold in grams (g) and the Hargreaves data are withdrawal latency in seconds (s). All other data are reported as duration of licking and biting in seconds (s). Where appropriate, we used two‐way ANOVA to compare effects of sex (male vs. female), genotype (ERα‐WT vs. ERα‐Cre; i.e., control vs. ablation), and their interaction. For the CFA behavioral test where the number of mice was not sufficient to analyze across the four groups, we pooled results from both sexes and performed a *t* test (or Mann–Whitney *U* test if groups were not normally distributed) to compare ERα‐WT versus ERα‐Cre (control vs. ablation).

**Figure 6:**
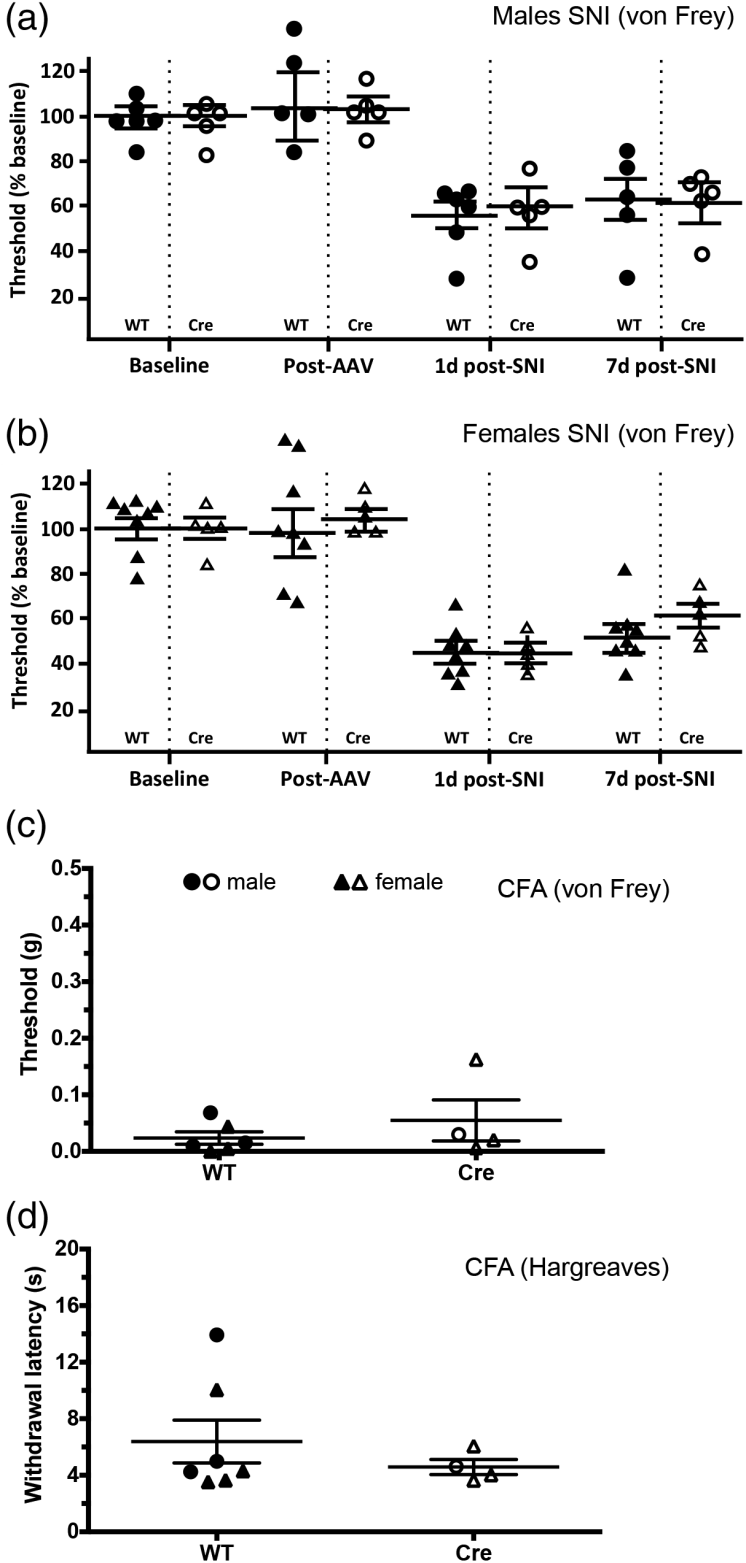
Lack of effect of spinal ERα^+^ neuron ablation in models of persistent pain. (a, b) One and 7 days after spared nerve injury (SNI), mechanical thresholds were tested in male (a) and female (b) ERα‐Cre mice and in their WT littermate controls. There was no significant difference between groups at either time point. (c, d) Three days after intraplantar injection of Complete Freund's Adjuvant, thresholds were tested in ERα‐Cre mice and WT littermate controls. Males and females were pooled due to low numbers. There was no significant difference between groups in mechanical allodynia (c) or thermal hyperalgesia (d). Data are presented as mean ± *SEM*

Lastly, in models of neuropathic (Figure 6a,b) and inflammatory (Figure 6c,d) persistent pain, we found no significant differences after ERα^+^ neuronal ablation. Note that in the CFA; Figure 6c‐d) model of chronic inflammatory pain, the number of mice with successful ERα^+^ cell ablation was insufficient for analysis by two‐way ANOVA. For this reason, we pooled male and female mice with successful ablation numbers and only compared by genotype. Using this approach (Table 2), there were no significant differences between groups in any of the tests or models examined.

## 4 DISCUSSION

In this study, we characterized a subpopulation of excitatory interneurons in the superficial spinal cord dorsal horn that express ERα subtype of estrogen receptor. Many of these neurons coexpress the nociceptive neuropeptide substance P, VGLUT2, and calretinin. After ERα^+^ neuronal ablation in adult mice, we observed sexually dimorphic deficits in the response to the chemical algogen, formalin, as well as reduced responses to the pruritogens, histamine, and chloroquine. Other nociceptive‐related behaviors in both naïve and tissue and nerve injury conditions were largely unaffected. Although we cannot exclude the possibility that the few ERα spinal neurons that survived the ablation procedure contributed to residual function, our results argue strongly that ERα marks a subpopulation of excitatory interneurons that are specifically involved in chemically evoked persistent pain and pruritogen‐induced itch.

### 4.1 Sex differences regulated by ERα‐expressing interneurons

Our finding that spinal ERα^+^ cells are primarily interneurons in lamina II agrees with a previous report (Amandusson et al., 1995). In addition, as reported by Vanderhorst Veronique et al. (2005), we observed comparable patterns and numbers of ERα expression in male and female mice. Although formalin appears to activate a male‐specific circuit that involves ERα^+^ interneurons, we suggest that instances of behavioral sexual dimorphism that we observed after ERα neuronal ablation likely reflect inputs to and outputs of these cells.

That there are sexual dimorphisms in nonreproductive signaling and behavior is well established—many drugs, including opioids, have different potencies in men and women and many drugs have been withdrawn due to an increase in adverse effects in women (Klein et al., 2015). Of course, such observations highlight the importance of performing experiments in both sexes. Unfortunately, due to concerns over fluctuating hormone levels in females during the estrous cycle, most studies are done exclusively in male subjects (Hughes, 2007). We did not control for estrous cycle differences in the present study, as a meta‐analysis of behavioral results in male and female mice suggests that this may not be critical (Mogil & Chanda, 2005). In fact, these authors reasoned that it is not necessary to control for the estrous cycle in female rodents because there are equally relevant fluctuations in male rodents, for example, changes in the dominance hierarchy. We recognize, however, that varying levels of estrogen during different estrous cycle stage may differentially affect the activity of ERα‐expressing spinal cord neurons in the female mice. For example, high levels of estrogen not only enhance temporomandibular joint (TMJ)‐evoked activity in superficial laminae (Okamoto, Thompson, Katagiri, & Bereiter, 2013) but also reduce morphine‐induced inhibition of these TMJ neurons (Okamoto, Tashiro, Hirata, & Bereiter, 2005; Tashiro, Okamoto, & Bereiter, 2008). Interestingly, the elevated sensitivity in von Frey mechanical threshold testing in females, compared to males, is consistent with reports by other groups (Mogil et al., 2006), and underscores the need to test subjects of both sexes, but whether it is essential to control for estrous status remains unclear.

### 4.2 Contribution of spinal ERα^+^ interneurons to nociceptive processing

We determined that the ERα‐expressing neurons are a subset of the population of spinal excitatory interneurons that are eliminated in the TR4‐Nestin knockout mouse (Wang et al., 2013), which shows an almost complete loss of the response to von Frey filament mechanical stimulation, capsaicin, formalin, chloroquine, and histamine. This mouse line also displays diminished or no mechanical allodynia following CFA or sciatic nerve injury (SNI). In the ERα^+^‐cell‐ablated mice, we detected similar defects, but in only two of the behaviors: the formalin test and pruritogen‐induced licking and biting. We conclude that ERα‐expressing interneurons contribute to a select portion of the TR4‐Nestin knockout phenotype, one that is mainly chemical. That conclusion is consistent with the report that formalin injection induces Fos expression in ERα‐interneurons (Amandusson & Blomqvist, 2010). It is likely that other populations of interneurons lost in TR4 mutant mice underlie the remaining behavioral abnormalities.

Previous studies reported that molecularly defined populations of dorsal horn interneurons mediate distinct modalities of pain and itch (Braz, Solorzano, Wang, & Basbaum, 2014). To date, four largely nonoverlapping neurochemical markers of adult excitatory interneurons have been described: substance P, neurokinin B, neurotensin, and gastrin‐releasing peptide (Gutierrez‐Mecinas et al., 2016; Gutierrez‐Mecinas et al., 2017). With the exception of gastrin‐releasing peptide, which is implicated in itch (Sun & Chen, 2007), it is not known whether specific pain or itch modalities, are associated with a particular excitatory interneuron subpopulation. We found that many, but not all, ERα^+^ cells, especially those in lamina II_o_, express substance P and/or calretinin, but not GRP. As substance P‐expressing neurons respond to a variety of algesic and pruritic stimuli (Gutierrez‐Mecinas et al., 2017), it appears that the ERα‐expressing neurons form a division within the substance P^+^ cells, one that is more specifically responsive to formalin and pruritogens.

Our demonstration of significant expression of substance P in the ERα interneurons is consistent with a recent analysis of the behavioral consequences of ablating dorsal horn Tac1‐Cre‐expressing interneurons (Huang et al., 2019). These authors reported that baseline reflexive responses to acute mechanical or thermal stimuli did not differ in the ablated and control mice, but that scratching‐evoked behaviors were significantly reduced in response to exogenous pruritogens. Interestingly, however, that report described a dramatic reduction of prolonged stimulus (e.g., burn injury)‐induced nocifensive behaviors and aversion, which the authors interpreted as a form of coping behavior. In light of our finding of sexually dimorphic behaviors in several chemically evoked pain and itch‐associated behaviors, it will be of interest to determine whether these coping behaviors are also sexually dimorphic. Finally, and as the majority of ERα‐expressing interneurons are excitatory, they presumably release glutamate. As substance P can potentiate glutamate‐induced currents in spinal dorsal horn neurons (Randić, Hećimović, & Ryu, 1990), it is conceivable that glutamate is co‐released by ERα‐expressing interneurons, allowing substance P to enhance the activity of glutamate and thereby strengthen synaptic connections and contribute to sensitization of dorsal horn neurons (Malmberg & Yaksh, 1992).

### 4.3 Estrogenic action on spinal ERα^+^ neurons

In a recent report, we identified a population of inhibitory dorsal horn interneurons that express aromatase, the enzyme that catalyzes conversion of androgens (e.g., testosterone) to estrogens (Tran et al., 2017). The aromatase‐expressing interneurons are concentrated in laminae I and V, placing them in close proximity to the ERα‐expressing interneurons, and of course, in regions intimately involved in the processing of pain and itch messages. Conceivably, activation of the aromatase interneurons concurrently inhibits the ERα‐expressing excitatory interneurons, providing an acute antinociceptive action, but via an estrogenic action, could provoke much longer term, possibly pro‐nociceptive, effects via downstream signaling pathways (Heldring et al., 2007). In fact, spinally synthesized estrogen has pro‐nociceptive effects. For example, in male Japanese quails, inhibition of local estrogen synthesis reduced responses to a noxious thermal stimulus (Evrard & Balthazart, 2004), while in male rats, inhibition of synthesis lowered pain scores in the formalin test (Zhang, Lü, Zhao, & Zhang, 2012). These results are consistent with the phenotypes that we observed after ERα^+^ cell ablation and suggest that by selectively knocking out ERα while preserving the neuron, future studies could more specifically address the contribution of estrogen to the activity of ERα‐expressing interneurons.

### 4.4 Conclusion

ERα is expressed by a subset of dorsal horn excitatory interneurons, many of which coexpress substance P. As our knowledge of nociceptive and pruriceptive circuitry develops, it has become increasingly clear that molecularly distinct categories of excitatory and inhibitory interneurons in the spinal cord define cell populations that convey different modalities of pain and itch. Functionally, the ERα‐expressing interneurons facilitate nociception, notably ongoing pain in the formalin model of postoperative pain, and pruritoception involving both histamine‐dependent and independent pathways. In addition to their involvement in acute chemonociception, the ERα‐expressing interneurons likely corelease substance P and glutamate to modulate the central sensitization that precipitates chronic pain states. Selective deletion of ERα from these interneurons, without affecting the rather extensive primary afferent, sensory neuron expression of the receptor, should provide answers to those questions.

## Data Availability

All raw data are presented in the Results section of the manuscript.
